# Prognostic factors in advanced pharyngeal and oral cavity cancer; significance of multimodality imaging in terms of 7th edition of TNM

**DOI:** 10.1186/1470-7330-14-15

**Published:** 2014-04-28

**Authors:** Maria Gődény

**Affiliations:** 1 Department of Radiolology, National Institute of Oncology, Budapest, Hungary; 2 Department of Postgraduate Education, Scientific Research of the University of Medicine and Pharmacy Tirgu Mures, Târgu Mureș, Romania

## Abstract

As with most cancers the prognosis in pharyngeal and oral cavity cancer largely depends on tumour stage. Physical examination, including endoscopy should be combined with technical radiologic imaging to record the precise extent of tumour. The TNM staging system of the head and neck region is, in fact, an anatomic staging system that describes the anatomic extent of the primary tumour as well as the involvement of regional lymph nodes and distant metastases. Modifications in the TNM staging system should consider not only the expert opinions and published reports in the literature but the technical advances in technology for improved assessment of tumour extent and the shifting paradigms in therapeutic strategies. “T” stage of the tumour is defined by its size, the depth of the invasion and the involvement of vital structures. In the 7th edition of TNM classification, for stage T4 tumors (larger than 4 cm), subcategories a and b were introduced to indicate the involvement of vital structures and their suitability for surgical resection (except for nasopharynx cancer). Nodal metastasis is the most important predictor of outcome for squamous cell cancer of the head and neck.

Better and more reliable methods of pretreatment tumour assessment are therefore crucial to ensure that the clinical assessment of tumor approximates its actual pathologic extent.

CT and MRI are both useful for assessing extensions of pharyngeal- and oral cavity cancer in advanced stage. MRI is superior in visualizing most primary tumour sites.

## Introduction

Hungary is first in pharyngeal- and oral cavity cancer (Ph-OC-CA) mortality in Europe in either sex. The male Ph-OC-Ca mortality is fourth in all cancer mortality in Hungary.

The majority of mucosal cancers in this country are squamous cell carcinomas (SCCA). Like in most cancers, the prognosis mainly depends on the stage of the tumour.

Head and neck (HN) is a difficult region, both its anatomy and pathology is very complex, various connection points may exist between the sites that determines the extension of disease.

Evaluation of Clinical Stage is based primarily on inspection, and palpation of the sites and on indirect and direct endoscopy. Neurologic evaluation of all cranial nerves is also required in advanced stage. Complete endoscopy, usually under general anesthesia, is performed after completion of other staging studies.

Imaging plays a crucial role in defining the extension of cancer and in therapy planning, as well. The accuracy of image evaluation depends very much on the experience and competence of radiologists. They have to be familiar with the anatomy and pathology of this region and the clinical details of the patient as well, in order to determine the most accurate stage of tumour.

## Review

### Basic aspects in the management of advanced head and neck cancer

There are new aspects in management of advanced Ph-OC-CA. One of the major endpoints of interest in management is to improve the quality of life, to preserve organ function without compromising survival. Chemotherapy and radiotherapy are considered as standards in treatment, but not all patients are suitable for organ preservation therapy. Patients with no response to standard therapy have to undergo salvage surgery. Criteria of inoperability have changed, consequently at present more extented resections and reconstructions are performed [1]. Tthe 3D-conformal-, intensity modulated radiotherapy (IMRT), for its better technical capacity, has resulted in a reduction of the dose delivered to normal tissues because of focusing more precisely on the target volumes. This means that more accurate assessment of tumour borders and tumour volume as well as of biological target volume is required in therapy planning [2].

Changes in treatment strategy of advanced HN cancer, necessitate more accurate radiological evaluation, knowledge of more tumour characteristics and prognostic factors.

It is crucial to ensure that the radiological diagnosis of the tumour status is concordant with the actual pathological status.

### Prognostic factors

Prognostic Factors affect patient’s survival and modify the therapeutic strategies. Tumour-related prognostic factors are: the primary tumour (T) size and its depth of invasion, the Grade of tumor, and the presence or absence of vascular invasion, − although the latter is particularly not included in the staging of HN tumours, but it should be noted when present [3,4]. The presence of metastatic lymph nodes (N), and their size, number, and position (level), as well as signs of extracapsular tumour spread (ECS**)** are important prognostic factors [5]. Mediastinal nodal spread is counted as distant metastasis, except those of level VII. The incidence of distant metastatic disease increases with rising tumour stage. Risk of distant metastasis is more dependent on “N” than on “T” status. As a rule, in Ph-OC-CA most metastases (M) develop in the lungs and the bones (rarely in the liver and brain). The data in the literature suggest that the rate of lung metastasis in newly diagnosed head and neck SCCA ranges from 16% to 19% using CT of the thorax [6,7]. Nasopharyngeal and adenoid cystic carcinomas (ACCA) have an increased risk of developing distant metastases.

In addition to the importance of the TNM factors, the overall health of these patients clearly influences the outcome. In some ongoing studies authors attempt to use both tumour and non-tumour related factors for the better evaluation of prognosis [4,8].

### Principle of the new, 7th TNM cancer staging

Numerous factors affect patient’s survival, including the histologic diagnosis, cellular differentiation of the tumour (grade), location and size, local extension, and the status of regional lymph nodes as well as the presence of distant metastases [9]. The importance of accurate mapping of the tumour before determining therapy has always been recognized by the Collaborative TNM System of the American Joint Committee on Cancer (AJCC) and the International Union for Cancer Control (UICC). The TNM system is periodically revised in response to newly acquired clinical data reflecting the new therapeutic strategies, improving understanding of cancer biology, and factors affecting prognosis. The revision cycle for TNM staging is 6–8 years. This provides sufficient time for the implementation of changes in clinical guidelines and cancer registry and for the discussion of data supporing changes in staging [3,4].

The 7”th TNM Revision is valid from 01, January, 2010 onwards. The anatomic extent of the disease remained the decisive factor in establishing prognosis in Ph-OC-CA.

The classification involves only four dominant clinical variables: tumour size, local extension of the tumour, nodal metastasis, and distant metastasis. Histologic grade, patient age and tumor site are important additional factors that should be recorded for future analysis and potential inclusion in the staging system.

ESMO Guidelines/2010 approved by the ESMO Guidelines Working Group, accepted the new therapy-guided 7th TNM strategies [1]. The guideline approved T4 tumors subdivision (except for nasopharyngeal cancer) into T4a: moderately advanced lesions and T4b: very advanced lesions with local extension. Clinical prognostic cancer stage IV was divided into: A, B, C subcategories. Stage IVA: moderately advanced (surgically resectable, suitable for salvage operation. Stage IVB: very advanced, “unresectable” are generally acknowledged as indicative of extension of tumor to vital anatomic structures, where surgical resection is either technically not feasible or not recommended however, it is potentially treatable with radio-chemotherapy. Stage IVC: stands for metastatic disease, only for palliative treatment: chemotherapy, radiotherapy.

### Role of imaging in clinical staging

Clinical staging, based on the best possible imaging technique, estimates the extent of disease before the first treatment. In the last ESMO Guidelines/2010 the diagnostic modalities in clinical staging were determined: physical examination, CT, MRI, HN-endoscopy and chest X-ray. According to the new EHNS-ESMO-ESTRO Clinical Practice: Guidlines for diagnosis, treatment and follow-up of the squamous cell carcinoma of the head and neck: MRI is considered the preferable staging procedure for every tumour subsite of the HN - except laryngeal and hypopharyngeal cancer [1]. MRI offers the best soft tissue differentiation, best evaluation of tumour borders, tumour extension, the most optimal evaluation of intracranial, perineural tumour spread, and also enables the radiologist to analyse vascularity. CT imaging with axial and coronal thin section technique using contrast agent is an excellant alternative diagnostic tool in Ph-OC-CA. In many institutions, CT is the preferred imaging method for the routine evaluation of head and neck cancer. In most cases a dedicated CT study will answer most questions of the referring clinician. CT is superior in the routine work to MRI: wide spread use, easy performance, standard reproducibility, relatively low cost, short examination time, resulting less motion artefact, superior information of cortical bone and bone lamellas without marrow space. CT can be regarded as the’workhorse’ of head and neck cancer imaging [10].

In advanced stage thoracic CT is preferable to exclude lung metastasis or second lung primaries [1,6,7].

3 T MR scanners can provide higher resolution images for better tissue definition and can also benefit from MR-spectroscopic imaging (MRSI) applications. MRI is a true cancer imaging biomarker. It may provide more physiologic and functional qualitative and quantitative data using dynamic contrast enhanced MRI (DCE-MRI), diffusion weighted MRI (DW-MRI) and can further improve the assessment of target volumes and can provide an opportunity to nominate the biological target volume and the potential to determine treatment response. The diagnostic accuracy of DWI complements anatomical MR imaging for staging neck lymph nodes in head and neck cancer (HNCA) [11-14].

The better estimation of target volumes with CT and MRI may permit treatment individualization [15]. The superior characterization of soft tissues and better visualization of tumour extent may be benefecial for radiotherapy planning because a more precise delineation of margins for the organ sparing strategies with boosting and dose escalation is feasible. The value of DW-MRI in radiotherapy planning is also under investigation. DW-MRI is able to characterize tissues and generate image contrast based on differences in histological micro-structures non-invasively, and based on water movement, improves the target delineation [11,13-16].

These techniques may also be combined with PET/CT to further increase diagnostic sensitivity and specificity. The role of FDG-PET/CT in staging is under investigation, it has a lower specificity than sensitivity and is more useful for staging distant metastases and synchronous tumour than lymphnode metastasis [1,17,18].

The need for extensive imaging to identify metastatic disease depends on the consideration of tumour site, stage of disease and histology. In advanced Ph-OC-CA a careful multimodality imaging search is mandatory also for other primary tumours of the upper aerodigestive tract and because of the higher incidence of multiple independent primary tumours occuring simultaneously [4].

US has been widely used in the assesment of cervical lymph node status, but it is not reliable for the deep nodes. US guided Fine-needle aspiration biopsy (USgFNAB) may confirm the tumour, its histopathologic nature, but it cannot rule out the presence of tumor [19].

### Key points in the evaluation of different Ph-OC-CA

#### Oral cavity cancers (OCCA)

The oral cavity is divided into the following specific sites:

Mucosal Lip (40% of OCCs), Buccal Mucosa, Lower Alveolar Ridge, Upper Alveolar Ridge, Retromolar Gingiva (Retromolar Trigone), Floor of the Mouth (15% of OCCs).

Hard Palate (5% of OCCs), Anterior two-thirds of the tongue (Oral Tongue, 20% of OCCs) The tongue is most commonly involved in the middle, and in the posterior third, mainly on the lateral, and lower surface. Human papilloma virus (HPV) DNA is prevalent in about 50% of oral cancer cases [10]. In OCCA submucosal spread is common. Tumours of each anatomic site have their own predictable patterns of regional spread [20]. The region of the retromolar trigone has important connection to the neighbouring cervical spaces, along the mandible to the pterygomandibular space and on this way to the parapharyngeal space till the skull base. Perineural spread may also occur [21].

The tongue is richly supplied with lymphatics, nearly half of patients have N metastases, 30%o of them are bilateral at the initial clinical presentation. Many of these nodes are clinically silent, detected only by imaging. Tumour thickness is also a prognostic factor that predicts subclinical nodal metastasis, local recurrence and patient survival [22].

**Figure 1:**
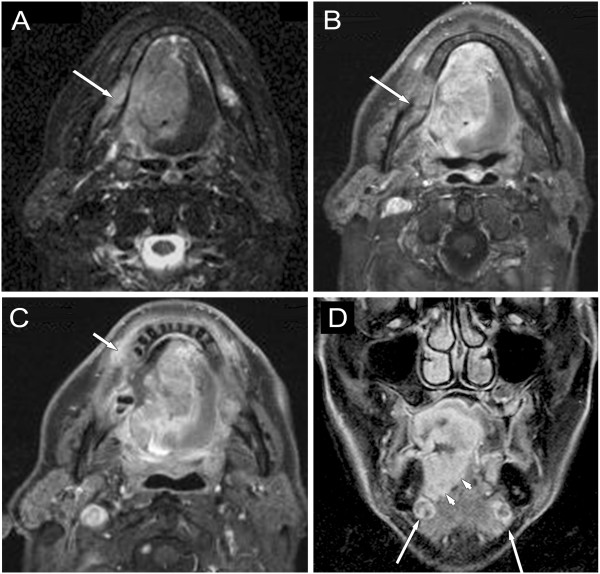
**Oral cavity cancer, located in the sublingual region, stage T4aN2c. A**: axial STIR-, **B**, **C**: axial post-contrast T1-weighted MRI images with fat suppression, **D**: coronal contrast-enhanced T1-weighted MRI images with fat suppression: demonstrate expansive carcinoma of the tongue infiltrating the gingiva (**C** image, short arrow), crossing the midline, invading the right mandibular base (**A**, **B** images, long arrows) and involving the extrinsic muscles of the tongue (**D** image, arrow-heads). On the coronal image bilateral small, well-defined submandibular lymph nodes are seen with sign of colliquation.

Clinical assessment of the extent of superficial mucosal involvement is more accurate than the radiographic assessment. MRI/CT are useful in evaluation of advanced tumours for the estimation of deep invasion (Figure 1). MRI is more accurate visualizing the extent of perivascular and perineural spread, skull base involvement, and intracranial tumour extension [20,21]. High-resolution CT images may provide additional information on bone and larynx details and they are minimally affected by motion artefact.

#### Key points in the’T’evaluation in advanced stages

•**T3** – TU more than 4 cm in greatest dimension

•**T4a – Moderately advanced local disease** : tumour invades the cortical bone, mandible, inferior alveolar nerve, deep/extrinsic muscle of tongue (genioglossus, hyoglossus, palatoglossus, styloglossus), maxillary sinus, skin of face

•**T4b - Very advanced local disease**: tumour invades the pterygoid plates, or skull base and/or encases internal carotid artery

Note: Superficial erosion alone of bone/tooth socket by gingival primary is not sufficient to classify T4. tumour [4].

### Nasopharyngeal cancer (NPhCA)

The primary tumour may be clinically occult and commonly present with neck nodal disease

**Figure 2:**
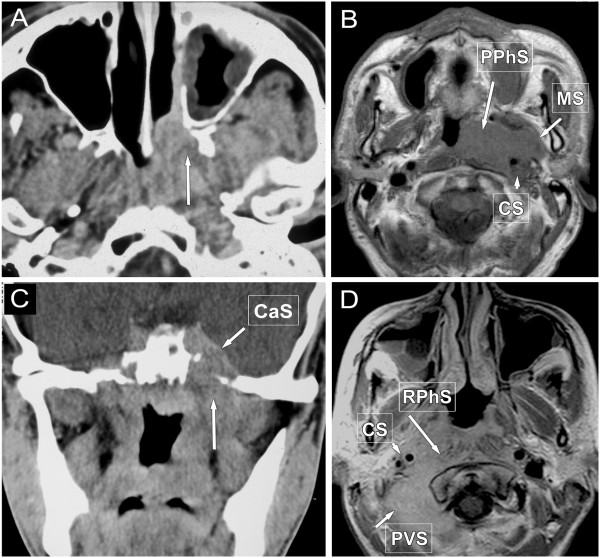
**Different patterns of spread in nasopharyngeal cancer. A**: Transverse post-contrast CT scan shows tumour destructing the left medial pterygoid lamina (long arrow), exceeding the choana and spreads along the lateral wall of the nasal cavity. Stage T3. **B**: contrast-enhanced T1-weighted MRI image demonstrates CA etxension to the parapharyngeal- (PPhS, long arrow), carotid- (CS, arrow-head) and masticator space (MS, short arrow) on the left side. Stage T4. **C**: Coronal contrast-enhanced CT image shows left sided destruction of the skull base (long arrow) and tumorous infiltration into the cavernous sinus (CaS, short arrow). Stage T4. **D**: Transverse post-contrast T1-weighted MR image demontrates tumour spread into the retropharyngeal space (RPhS, long arrow) on the right side, infiltrating the longus colli muscle, involving also the carotid- (CS, arrow-head), and paravertebral space (PVS, short arrow). Stage T4.

**Figure 3:**
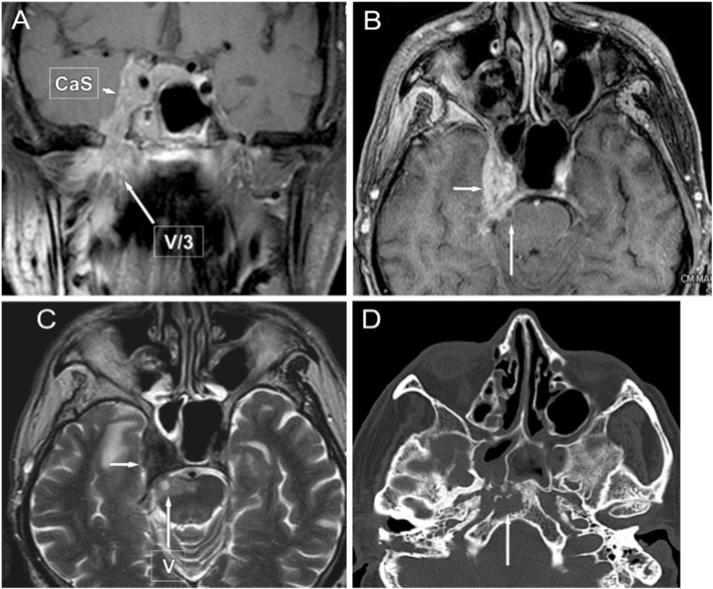
**Extensive nasopharyngeal cancer with skull base destruction and perineural spread into the brainstem. (T4). A**, **B**: coronal and axial post-contrast T1-weighted MR image with fat suppression. **A** image shows tumor extension from the nasopharynx through the right wide foramen ovale and perineural spread along the V/3 (long arrow) into the cavernous sinus (CaS, arrow-head). The CaS is flared out towards the Meckel’s cavity and tumorous enlargement of the trigeminal ganglion (semilunar, or Gasser) can be well seen (on **B**, **C** images, short arrows). **C**: axial T2-weighted image shows signal intensity changes in the pons secondary to the perineural tumour spread along trigeminal nerve root (on **B**, **C** images, long arrows) **D**: axial nonenhanced CT scans show tumorous bone destruction of the clivus on the right side (long arrow).

Most NPhCA arise in the lateral pharyngeal recess (fossa of Rosenmüller) and spread deeply, often obsruct the eustachian tube. The first two tumour stages are redefined in the 7th TNM: Tumour is T1 stage not only when confined to NPh, but extending to oropharynx and/or nasal cavity without parapharyngeal extension and T2 stage is only for parapharyngeal tumour extension. Primary NPhCA commonly spreads to the deep neck space and also intracranially (Figures 2 and 3). Majority (75%) [23] of NPhCA patients have cervical N metastases at presentation, with bilateral involvement in up to 80%. Retropharyngeal (RP) lymphnodes are the first lymphatic filters, regardless of unilateral or bilateral location, are considered N1 [4], and can be better evaluated on MR than on CT. 35% of metastases bypass the retropharyngeal nodes and the tumour appears first in the level II nodes being the first echelon, although the upper jugular, and spinal accessory nodes are considered second filters.

Nodes may be also clinically silent, and detectable by MRI/CT. MR provides better delineation of RP nodes from an adjacent primary tumour. CT is rarely used to look at bone invasion if MRI is available. In the vast majority of the cases the management of NPhCA is confined to radiotherapy.

#### Key points in the’T’ evaluation in advanced stages

•**T3** – tumour invades bony structures of skull base and/or paranasal sinuses

•**T4** – tumour with intracranial extension and/or involvement of cranial nerves, hypopharynx, orbit, extension to the infratemporal fossa/masticator space

### Oropharyngeal cancer (OPhCA)

The OPh includes the following specific sites:

The base of the tongue, the inferior (aterior) surface of the soft palate and the uvula, the anterior and posterior tonsillar pillars, the glossotonsillar sulci, the pharyngeal tonsils, and the lateral and posterior pharyngeal walls.

The human papillomavirus (HPV) status of a tumour is a strong and independent prognostic factor for survival in patients with cancers of the oropharynx- and oral cavity CA. Studies demonstrated that high HPV prevalence has been consistently demonstrated in OCCA/OPhCA compared to a lower prevalence in other HN-SCCAs. HPV positive tumours were observed in younger, lighter alcohol-consuming patients with a better overall and disease specific survival [24].

**Figure 4:**
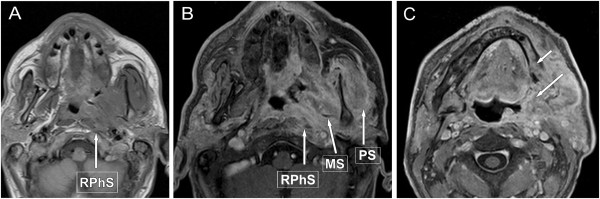
**Oropharyngeal cancer, stage T4b. A**: axial, non-enhanced T1-weighted, **B**, **C**: axial post-contrast T1-weighted MR images with fat suppression show tumour involving the lateral wall of the oropharynx on the left side, widely extending towards the retromolar trigone of mandibule (**C** image, long arrow), destructing the mandibular cortex, involving the marrow space (**C** image, short arrow). Note the extensive tumor involvement (long arrows) of the retroharyngeal- (RPhS), carotid-, masticator- (MS), as well as the parotid space (PS).

**Figure 5:**
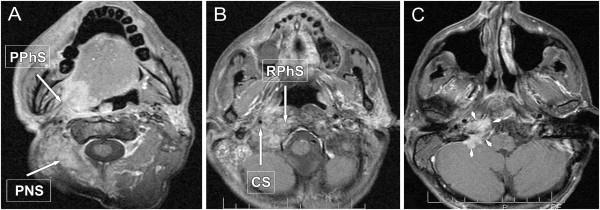
**Oropharyngeal cancer, arising from the base of the tongue on the right side, stage T4b A, B, C: axial post-contrast T1-weighted MR images with fat suppression.** The estimated tumour stage by clinical examination was T2, but note the tumor extension in the deep cervical structures, trough the parapharyngeal- (PPhS), carotid- (CS), retropharyngeal-(RPhS) and posterior neck space (PNS, long arrows) into the scala posterior, involving the apex of petrous part of temporal bone and pontocerebellar region (arrowheads).

Also in OPhCA submucosal spread is common (Figure 4). Intracranial tumor extension may also occur through the deep neck spaces (Figure 5).

The most common sites of lymphnode metastasis are the upper- (Level II) and mid-jugular- (Level III) and less commonly submental/submandibular (Level I) regions [4,10,25].

#### Key points in the’T’ evaluation in advanced stages

•**T3** – tumour >4 cm/extension to lingual surface of epiglottis

•**T4a** – **Moderately advanced local disease:** tumour invades: larynx, extrinsic muscle of tongue, musculus pterygoideus medialis, hard palate, mandible

•**T4b** - **Very advanced local disease**: tumour invades: musculus pterygoideus lateralis, pterygoid plates, NPh, skull base, prevertabral fascia, encases the carotid artery

Mucosal extension to lingual surface of epiglottis from primary tumours of the base of tongue and vallecula does not constitute invasion of larynx [4].

### Hypopharyngeal cancer (HPhCA)

**Figure 6:**
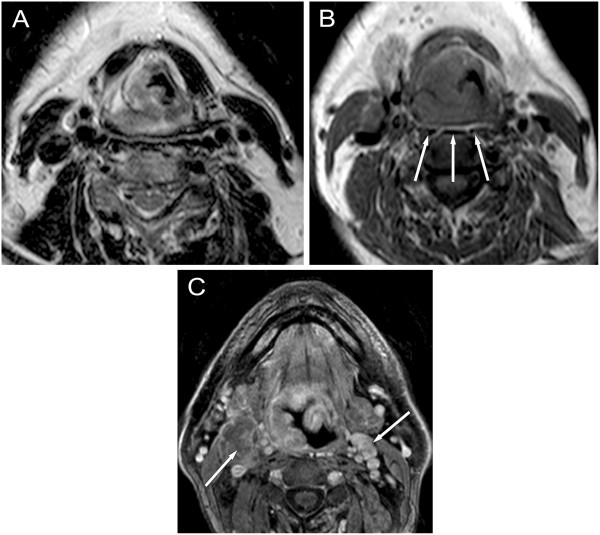
**Hypopharyngeal cancer, stage T4aN2c. A**: axial T2-weighted, **B**: axial non-enhanced T1-weighted MR images show cancer of the hypopharynx involving the dorsal and right lateral pharyngeal wall, avoiding the retropharyngeal space. Note the intact prevertebral fat, clearly obtainable on T1-weighted image (arrow). **C**: axial post-contrast T1-weighted MR image with fat suppression at the level of the tongue base, tumour infiltrates the tongue base crossing the midline to the opposite side. Metastatic lymph nodes are present bilaterally (arrows).

**Figure 7:**
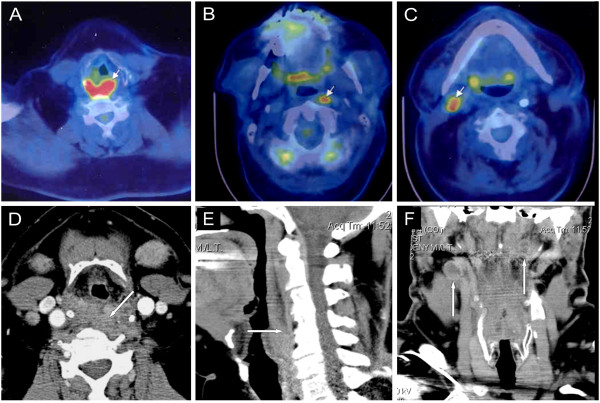
**Hypopharyngeal cancer with bilateral metastatic lymph nodes - was performed to detect the site of an unknown primary tumour.** On axial plane PET/CT images: **A**, **B**, **C**, the examination revealed cancer on the dorsal pharyngeal wall (arrow), and suspected the presence of metastatic lymph nodes in the deep cervical lymphatic regions bilaterally (arrows). Multiplanar Contrast-enhanced-CT images: **D**, **E**, **F** scans in axial, sagittal and coronal planes also demonstrate a mass in the dorsal wall of the hypopharynx, that affects the integrity of the fat layer in the prevertebral space, clearly detectable on sagittal plane image (arrow). Coronal CT scan shows metastatic lymph nodes bilateraly (arrows) with colliquation, however they are less detectable as by PET/CT. Estimated stage by CT: T4b N2c.

**Figure 8:**
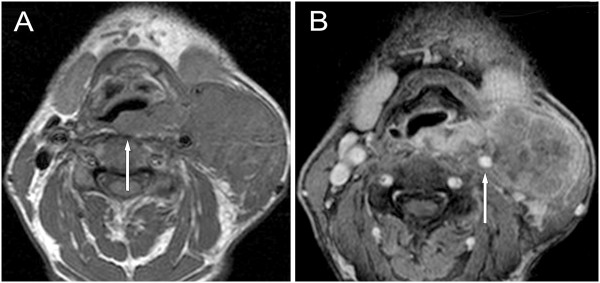
**Hypopharyngeal cancer, stage T4bN3. A**: transverse non-contrast T1-weighted, **B**: transverse post-contrast T1-weighted MR images with fat suppression demonstrate cancer of the dorsal hypopharynx wall passing through the retropharyngeal fascia disrupting the prevertebral fat plane (**A** image, arrow). A large 7 cm in diameter nodal conglomerate is visible on the left side, connected to the tumour. The primary tumour and the connected conglomerate encircles the internal carotid artery (**B** image, arrow), resulting an inoperable condition. The patient recieved radiochemotherapy.

Primary HPhCA may remain asymptomatic for a long period but at presentation the disease is often advanced commonly presenting with neck N disease (50-70%) [4,10,25]. The tumour volume significantly influences patient’s prognosis. HPhCA is commonly infiltrating into the surrounding structures rather than suspected on clinical grounds [20]. Cancer of the posterior pharyngeal wall commonly appears as a flat but often widely extended lesion that may invade the retropharyngeal-prevertebral fascia. The absence of prevertebral space involvement is reliably predicted on CT/MRI by demonstrating the preservation of the retropharyngeal fat plane [26] (Figure 6). Those cases extended into the prevertebral space are categorized as T4b, because the likelihood of curative surgical resection is minimal, if any, therefore such patients are treated by non-surgical modalities (Figures 7 and 8). However, CT/MRI are less accurate in predicting the involvement of the prevertebral space, in these findings the positive predictive values are lower than the negative ones.

Bilateral lymphatic drainage is common. HPhCA spreads to adjacent parapharyngeal, jugular, paratracheal nodes: Levels: II, III, IV, VI and VII.

#### Key points in the’T’ evaluation in advanced stages

•**T3** – tumour more than 4 cm in greatest dimension, or with fixation of hemilarynx, or extension to esophagus

•**T4a** – **Moderately advanced local disease,** tumour invades: thyroid-/cricoid cartilage, hyoid bone, thyroid gland, or central compartment soft tissue (central compartment soft tissue includes prelaryngeal strap muscles and subcutaneous fat)

•**T4b** – **Very advanced local disease**, tumour invades retropharyngeal/prevertebral fascia, encases carotid artery, or involves mediastinal structures

### Key points in the evaluation of the neck lymphnode status

The presence of neck adenopathies influences patient management, and largely determines the chance for locoregional control and the risk for distant metastasis. The risk of N metastasis is generally related to the depth of infiltration of the tumour.

**Figure 9:**
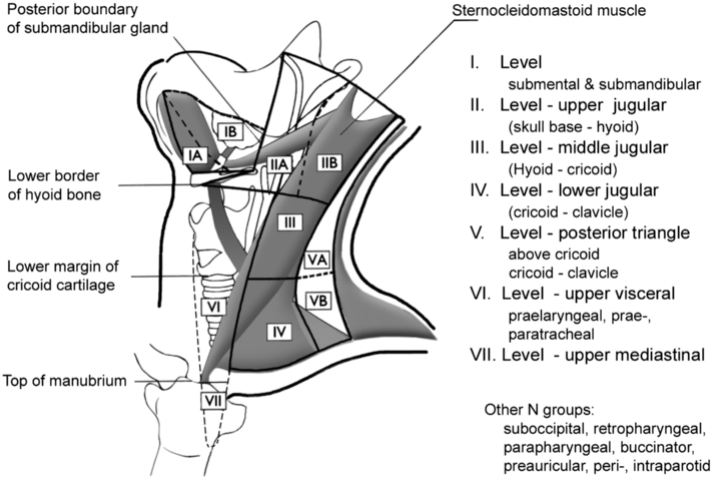
**Cervical lymph node regions - Imaging-Based Level System *(Som).***

Nodal disease can be treated either surgically, by performing a neck dissection, or by chemo-radiotherapy. Occurrence of N metastasis decreases the overall survival to its half [27]. Tumours of each anatomic site have their own regional lymphatic spread and different metastatic potential. The lip-, hard palate-, alveolar ridge-, glottic, sinonasal cancers have low metastatic potential, but the other oral-, nasopharyngeal, meso-, hypopharyngeal, supraglottic cancers are in high risk metastatic group [4]. In the high risk groups cancers spread primarily to the first lymphatic filter and to the lower level nodes, occasionally spread directly to the lower nodes. To facilitate communication, radiologists should be familiar with the anatomical distribution of cervical nodes (Figure 9) used by surgeon as well as pathologists [28].

•**Nasopharyngeal cancer:** commonly spreads to **retropharyngeal,** upper jugular **(Level II)** and spinal accessory (**Level V)** nodes, often bilaterally. NPhCA with retropharyngeal node involvement is N1 stage - independent of laterality and without cervical node involvement (it is new in the 7th TNM)

•**Oral cavity cancer:** commonly spreads to submental, submandibular **(Level I),** internal jogular **(Level II)** nodes.

•**Oropharyngeal cancer:** involves upper and mid-jugular nodes (**Level II, III**) and (less commonly) submental/submandibular nodes **(Level I**)

•**Hypopharyngeal cancer:** commonly spreads to adjacent parapharyngeal, paratracheal **(Level VI),** and mid- and lower jugular nodes **(Level III, IV).** Bilateral lymphatic drainage is common.

**Figure 10:**
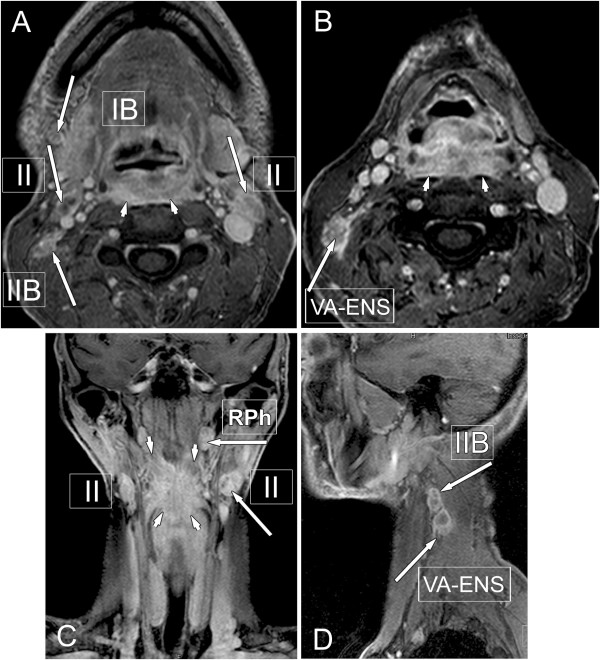
**Metastatic lymph nodes. A**, **B**, **C**, **D** figures: post-contrast T1-weighted MRI images with fat suppression showing carcinoma of the dorsal pharyngeal wall (arrow-heads) with signs of retropharyngeal (RPh) tumor spread manifesting in bilateral retropharyngeal lymphnode enlargement above the upper level of tumour (large arrow on “**C**” image). Long arrows show metastatic lymph nodes in different cervical regions, with necrotic center, and irregular margins as typical sign of extranodal tumour spread (ENS).

The level of involved nodes in the neck is prognostically significant, lower is worse, as is the size above 6 cm and presence of extranodal spread (ENS). Secondary to the ENS involvement of the carotid arteries, it has bad prognostic and therapeutic relevance [4,27] (Figure 10). The patterns of regional N metastasis are predictable, and the sequential progression of disease occurs beyond the first echelon lymph nodes.

The cervical lymphnode classification for metastasis is uniform for all sites, except thyroid, nasopharynx, and skin. Node size, number and location are prognostic factors. Masses ≥ 3 cm are rarely single lymphnodes, but rather fusion of metastatic nodes.

•**N1:** single N ≤ 3 cm,

•**N2a:** single N ipsilateral > 3 cm ≤ 6 cm,

•**N2b:** multiple ipsilateral ≤ 6 cm,

•**N2c:** bilateral or contralateral N ≤ 6 cm,

•**N3:** > 6 cm.

(The lymphnode size should be measured in greatest dimension.)

Midline N are considered ipsilateral N. The closer to the midline the tumour, the greater the risk of bilateral N metastasis is. Mediastinal N metastases are distant metastases, but superior mediastinal N are considered regional N (level VII) [3,4].

### Role of imaging to determine N status

Cervical lymphadenopathy is one of the most important key points of Ph-OC-CA. The sensivity (Sv) and specificity (Sp) of palpation for neck N metastasis are in the range of 60%-70% [28]. Clinical evaluation of neck lymph nodes is not very precise, subclinical adenopathies may be detected by imaging studies [27]. With US more nodes can be inspected than with the clinical examinations, but CT/MR/PET-CT are more accurate than US. Particular attention should be paid to clinically inaccessible nodal sites such as the retropharyngeal and paratracheal lymph nodes.

The ***radiological criteria of metastasis*** are based not only on increased N size (>1 cm), and round shape but rather on the presence of tumour colliquation, and tumour extension beyond the margin of the node. In the size criterion, the minimal axial diameter is more decisive than the other diameters [29]. The inhomogeneous nodal structure, the signs of central necrosis, and the signs of the extranodal tumour spread, the spiculated margins, involvement of internodal fat, and loss of normal oval-round nodal shape distinctly appear on both CT and MRI and are typical signs for metastatic nodes. Both CT and MRI have accuracy (Acc) of 73%-80% to detect ENS [18,29-31].

US is superior to palpation and has been widely used in the assesment of cervical lymphnode status, but it is not reliable for deep nodes (e.g., retropharyngeal nodes) because of its limited penetration into deeper layers. In addition, US is highly dependent on the experience of the operator. US is relatively cheap and capable of guiding tissue sampling. The USgFNAC has very high Sp, approaching 98-100% with a Sv of 73-80% [31], but this method is time consuming, and operator dependent [10,19].

A meta-analysis comparing CT with physical examination (PE) yielded the following results: Sv, 83% (CT) vs 74% (PE); Sp 83% (CT) vs 81% (PE); and Acc, 83% (CT) vs 77% (PE). Overall, PE identified 75% of patologic cervical adenopathies. This detection rate increased to 91% with addition of CT [10,32].

According to another analysis, the Acc of CT and MR in staging N involvement is almost similar (73-80%) [30]. The advantage of these techniques is the standard, comprehensive evaluation of the entire head and neck region, also allowing detection of retropharangeal adenopathies [19,28-30].

A tumour encircling the vessel over 270° on CT or MRI, or a tumour that is immobile from the vessel using sonopalpation, confirms the involvement of the vessel wall and is often non-resecable. With real time US can be helpful to detect carotid wall invasion [33,34].

***MR-lymphography*** for N staging, using superparamagnetic iron oxid particles as contrast agents, may increase accuracy, Sv of 86% and Sp of 100% for metastatic nodes, but micrometastases may be missed also with this method [35]. (Disadvantage: MR-lymphangiographic agents are not commercially available at present)

***Sentinel node biopsy*** is widely accepted as the standard practice in the management of breast cancer, cutaneous malignant melanoma and the procedure was also under investigation in HN-SCCA but with less accuracy than in the above mentioned tumours [36-38].

***PET-CT*** can map functional and metabolic activity but less accurately can identify tumour in normal sized lymph nodes. Also the false negative necrotic nodes are potential pitfalls in the analysis of PET-CT. FDG-PET/CT has a lower specificity than sensitivity and more useful for detecting distant metastases and synchronous tumour than lymphnode metastasis [1,17,18].

**Figure 11:**
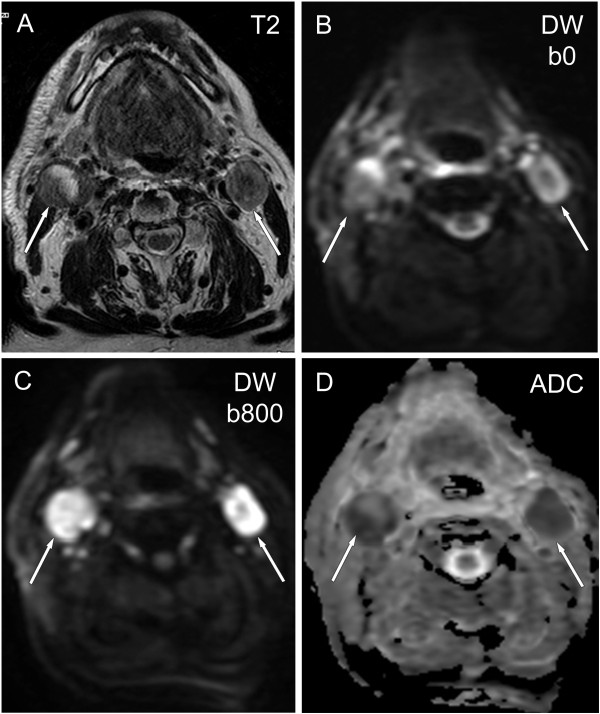
**Irradiated oropharyngeal cancer with residual lymphnode enlargement (arrows). A**: axial TSE T2-weighted image shows heterogeneous enlargement of the irradiated lymphnodes. **B**, **C**, **D**: Diffusion weighted images in transverse plane demonstrate inhomogeneous restricted diffusion with increased signal on DW-images (**B**: b value of 50 s/mm^2^, **C**: b value of 800 s/mm^2^) and decreased signal on ADC map **(D)** corresponding residual tumour in the nodes.

Early experience with ***DW-MRI,*** indicates that a significantly higher sensivity may be attained [2,11-14,39]. With an optimal ADC _b0–1000_ threshold of Sv 84%, Sp 94%, and Acc 91% for differentiation of malignant versus benign status of each lymphnode and Sv 94%, Sp 97%, and Acc 97% for differentiation at each neck level were achieved (Figure 11). Compared with turbo spin-echo imaging, DW-MRI had higher Sv (76% vs. 7%) but slightly lower Sp (94.0% vs 99.5%) in detecting subcentimeter N metastases [14].

None of the currently available imaging methods, including FDG-PET/CT, are reliable to detect small tumour deposits within non-enlarged lymph nodes, or differentiate reactively enlarged lymph nodes from metastatic lymphadenopathy. Patients with stage N0 still undergo elective nodal resection for diagnostic staging and 20-25% of the patients have metastatic nodes in pathology [27].

## Conclusion

Accurate and reliable stratification of Ph-OC-CAs for the prediction of outcomes has been challenging, mainly because of the numerous anatomic sites and subsites from which tumours can arise and the diversity of histologic types of tumours in these locations.

Accuracy of pretreatment staging has become more critical after the introduction of “organ preservation” protocols, since non-surgical therapy and the very extended, more invasive salvage surgery have become widely accepted treatment possibilities.

“T” stage of the tumour is defined by its size, the depth of the invasion and the involvement of vital structures. Tumours that invade the prevertebral fascia, skull base, cranial nerves or encase the carotid or mediastinal great vessels are not resecable, are qualified as Stage T4b, according to the latest TNM classification.

In most cases treatment outcome is strongly influenced by the presence or absence of cervical nodal metastasis. Nodal necrosis and extracapsular spread indicates metastatic disease, irrespectively of nodal size.

Imaging plays an important role in clinical evaluation of head and neck lesions. A particular advantage of cross-sectional imaging is the ability to detect deep tumor extension or the presence or absence of metastases that cannot be judged by clinical examination. “Stage migration” occurs when patients are assigned to different clinical stages because of differences in the accuracy of the clinical examinations and imaging staging methods. Compared with conventional clinical examination, MRI and CT render a more precise assessment of tumour possible that may lead to the classification of the tumour into a higher clinical stage.

CT/MR are also mandatory in selecting patients for a favourable group for radiotherapy by providing an estimate of tumour borders and volume.

The radiologist’s task is to determine the site, extension and volume of the disease, to differentiate tumour from inflammation. Imaging can help oncologists, surgeons, radiotherapeutists better determine the location of tumours, confirm or exclude suspected diseases, avoid the complications, evaluate the clinical key points, and also detect the endoscopic ‘blind spots’ in clinically occult cases.

The accuracy of image evaluation depends highly on the experience and competence of radiologists. The radiologist plays an indisputable role in the Ph-OC-CA multidisciplinary team and has to be strongly committed to the management of patients.

## Competing interests

The author declares that she has no competing interests.
